# Circulating Tumor Cells (CTC) and Cell-Free DNA (cfDNA) Workshop 2016: Scientific Opportunities and Logistics for Cancer Clinical Trial Incorporation

**DOI:** 10.3390/ijms17091505

**Published:** 2016-09-08

**Authors:** Lori E. Lowes, Scott V. Bratman, Ryan Dittamore, Susan Done, Shana O. Kelley, Sabine Mai, Ryan D. Morin, Alexander W. Wyatt, Alison L. Allan

**Affiliations:** 1 London Regional Cancer Program, London Health Sciences Centre, London, ON N6K 4L6, Canada; Lori.Lowes@lhsc.on.ca; 2 Special Hematology/Flow Cytometry, London Health Sciences Centre, London, ON N6K 4L6, Canada; 3 Departments of Radiation Oncology and Medical Biophysics, University of Toronto, Toronto, ON M5G 1L7, Canada; Scott.Bratman@rmp.uhn.ca; 4 Princess Margaret Cancer Centre, University Health Network, Toronto, ON M5G 1L7, Canada; 5 Epic Sciences Inc., San Diego, CA 92121, USA; ryan.dittamore@epicsciences.com; 6 Campbell Family Institute for Breast Cancer Research and Laboratory Medicine Program, University Health Network, Toronto, ON M5G 2C4, Canada; Susan.Done@uhn.ca; 7 Departments of Laboratory Medicine and Pathobiology, and Medical Biophysics, Faculty of Medicine, University of Toronto, Toronto, ON M5G 2C4, Canada; 8 Department of Pharmaceutical Sciences, Leslie Dan Faculty of Pharmacy, University of Toronto, Toronto, ON M5S 3M2, Canada; shana.kelley@utoronto.ca; 9 Department of Biochemistry, Faculty of Medicine, University of Toronto, Toronto, ON M5S 3M2, Canada; 10 Department of Chemistry, Faculty of Arts and Science, University of Toronto, Toronto, ON M5S 3M2, Canada; 11 Manitoba Institute of Cell Biology, Cancer Care Manitoba, University of Manitoba, Winnipeg, MB R3E 0V9, Canada; Sabine.Mai@umanitoba.ca; 12 Department of Molecular Biology and Biochemistry, Simon Fraser University, Burnaby, BC V5A 1S6, Canada; rdmorin@sfu.ca; 13 Vancouver Prostate Centre and Department of Urologic Sciences, University of British Columbia, Vancouver, BC V6H 3Z6, Canada; awyatt@prostatecentre.com; 14 Departments of Anatomy & Cell Biology and Oncology, Schulich School of Medicine and Dentistry, University of Western Ontario, London, ON N6K 4L6, Canada

**Keywords:** oncology, circulating tumor cells (CTC), cell-free DNA (cfDNA), circulating tumor DNA (ctDNA), clinical trials, conference report

## Abstract

Despite the identification of circulating tumor cells (CTCs) and cell-free DNA (cfDNA) as potential blood-based biomarkers capable of providing prognostic and predictive information in cancer, they have not been incorporated into routine clinical practice. This resistance is due in part to technological limitations hampering CTC and cfDNA analysis, as well as a limited understanding of precisely how to interpret emergent biomarkers across various disease stages and tumor types. In recognition of these challenges, a group of researchers and clinicians focused on blood-based biomarker development met at the Canadian Cancer Trials Group (CCTG) Spring Meeting in Toronto, Canada on 29 April 2016 for a workshop discussing novel CTC/cfDNA technologies, interpretation of data obtained from CTCs versus cfDNA, challenges regarding disease evolution and heterogeneity, and logistical considerations for incorporation of CTCs/cfDNA into clinical trials, and ultimately into routine clinical use. The objectives of this workshop included discussion of the current barriers to clinical implementation and recent progress made in the field, as well as fueling meaningful collaborations and partnerships between researchers and clinicians. We anticipate that the considerations highlighted at this workshop will lead to advances in both basic and translational research and will ultimately impact patient management strategies and patient outcomes.

## 1. Introduction

The vast majority of cancer-related deaths are due to the spread of disease from the primary site to distant sites throughout the body, through a process known as metastasis. This stage of disease presents a number of challenges to treating physicians due to the highly aggressive and treatment-resistant nature of metastases, as well as limitations with regards to monitoring disease progression over time [1,2]. Although the field of oncology has seen significant improvements in the treatment of this disease over the last 25 years [3], challenges still exist with regards to patient stratification (i.e., determining which patients are high- versus low-risk) and in monitoring treatment efficacy [4]. With these challenges in mind, the field has identified a need for the development and validation of novel biomarkers that could provide physicians with additional prognostic and/or predictive information. This information could then be utilized to aid in clinical-decision making and ultimately improve patient outcomes.

Over the past decade, circulating tumor cells (CTCs) and cell-free DNA (cfDNA; also known as circulating tumor DNA (ctDNA) when believed to have originated from a tumor cell) have been identified as potential blood-based biomarkers capable of providing such information [5]. However, implementing these valuable tools into widespread clinical use has proven to be more difficult than originally anticipated, due in part to a limited understanding of how these biomarkers should be interpreted across various disease stages and tumor types, as well as technological limitations regarding CTC and cfDNA detection and analysis [5,6]. It is these limitations that led to a gathering of CTC and cfDNA researchers and clinicians at the Canadian Cancer Trials Group (CCTG) Spring Meeting in Toronto, Canada on 29 April 2016 for a workshop focused on the scientific opportunities and methodological and logistical challenges for incorporation of CTCs and cfDNA into clinical trials, and ultimately into routine clinical use.

The objectives of the Workshop, Co-Chaired by Alison Allan (University of Western Ontario, London, ON, Canada) and Alexander Wyatt (University of British Columbia, Vancouver, BC, Canada) were:

(1) To highlight Canadian work in the areas of CTCs and cfDNA;
(2) To provide an update on current technologies including advantages and limitations, potential clinical utility for analysis of CTCs and cfDNA, and recent translational breakthroughs;
(3) To consider optimal clinical trial designs for CTC and cfDNA incorporation, including discussion of the best standard operating procedures (SOPs) for collection and analysis; and
(4) To provide networking opportunities for clinical and translational researchers, and facilitate new collaborations in the area of CTCs/cfDNA and cancer clinical trials.

During the Workshop, presentations by speakers and the resulting interactive discussion with workshop attendees highlighted a number of important themes and considerations, and these are summarized in this Report. For an extensive overview of CTCs and cfDNA please refer to a number of excellent recently published review articles [5,7,8].

## 2. Theme 1: Technological and Methodological Advances in Circulating Tumor Cells (CTCs) and Cell-Free DNA (cfDNA) Detection and Analysis

The field of CTC and cfDNA research has recently seen an explosion in the number of technologies available for both capture and analysis of these rare biomarkers. However, with the exception of the CellSearch^®^ CTC platform (Janssen Diagnostics, Raritan, NJ, USA) and a few other emerging systems, many CTC and cfDNA technologies face a general lack of standardization, a necessity for entrance into the clinical setting. This is particularly evident when considering cfDNA, for which only one clinically validated companion diagnostic test currently exists, the cobas^®^ EGFR Mutation Test v2 (Roche Molecular Systems, Pleasanton, CA, USA) for use specifically in predicting response to Tarceva (erlotinib) in non-small cell lung cancers [9].

Technologies aimed at CTC enrichment have grown from very basic, single-sample manual approaches to fully automated platforms capable of processing many samples simultaneously. These available technologies have been extensively reviewed previously [10] and therefore will not be discussed in detail here. In brief, CTC isolation and detection is usually a two-step process, involving both an enrichment step (using size-, density-, immunomagnetic-, or microfluidic-based approaches), and a detection/characterization step (typically protein-, or nucleic acid-based). Although developed and validated over 10 years ago, the CellSearch^®^ system (Janssen, Raritan, NJ, USA) still remains the gold standard CTC platform in the clinical setting [11]. This laboratory “workhorse” is currently the only CTC platform with Food and Drug Administration (FDA) and Health Canada clearance for in vitro diagnostic use in metastatic breast, prostate, and colorectal cancers [11,12,13]. The rigorous clinical data generated using this platform and its observed strengths (i.e., reproducibility, validated standardization, demonstrated prognostic relevance in the metastatic setting) and limitations (e.g., closed user platform with limited capture and characterization capabilities, low sensitivity in non-metastatic settings) have influenced our interpretation of CTC findings using other instrumentation (i.e., providing appropriate prognostic “cut-off” values to use as starting points for technology validation (≥5 CTCs for breast and prostate cancer; ≥3 CTCs for colorectal cancer)), as well as the development of second- and third-generation technologies.

Within the cfDNA realm, fetal cfDNA screening has been a cornerstone of pre-natal diagnoses for years, but has been under-recognized and under-exploited in oncology due to technology limitations and associated costs. Although there have not been significant changes in approaches for isolation of cfDNA from whole blood since the advent of the field over 20 years ago [14,15,16], improvements in products used for blood collection/storage [17] and the sensitivity/specificity of downstream analysis approaches (e.g., next-generation sequencing (NGS), digital droplet PCR, and BEAMing (beads, emulsion, amplification, magnetics) PCR) have changed dramatically. Additionally, the increased speed of sample analysis as well as the reduction in processing costs has made cfDNA a viable candidate for integration into the clinical setting [6,10]. However, as mentioned previously, one major challenge facing the integration of cfDNA into routine clinical practice is limited pre-analytical and analytical SOPs, assay validation, and identification of appropriate prognostic/predictive read-out (e.g., concentration of cfDNA, presence/absence of specific mutations). It is also important to note the cfDNA field is further complicated by the fact that circulating DNA (which may originate from either tumor cell or other cell types (e.g., leukocytes)) must be appropriately differentiated from circulating tumor DNA [6]. The origin of cfDNA can typically be determined based on detection of specific known target mutations, which may vary between disease type and stage. This complexity further exemplifies the importance of appropriate assay design, validation, and standardization. Additional challenges exist with regards to using cfDNA for the competing concepts of diagnosis versus patient monitoring. It is highly probable that these technologies will need to differ considerably in order to obtain the necessary information.

The CTC/cfDNA technology industry is crowded, making technology selection a confusing process. In fact, even the clinically-used CellSearch^®^ system has been criticized for its reliance on the widely utilized antigens EpCAM and cytokeratin for CTC isolation, as these antigens have been demonstrated to be down-regulated during the cellular processes that allow for cancer cell invasion into the bloodstream [18]. This confusion is further amplified when considering the overwhelming number of companies focused not on isolation of CTCs or cfDNA but on the downstream analysis of these rare events. This is especially prevalent for cfDNA in which there is significant competition amongst commercial platforms for digital PCR technologies (e.g., BioRad’s QX200 Droplet Digital™ PCR system (Hercules, CA, USA), and RainDance Technologies’ RainDrop™ Digital PCR system (Billerica, MA, USA)). Therefore it is not surprising that the speakers at this CTC/cfDNA Workshop presented data and observations from a variety of different technologies and approaches, based on their needs and available resources, with some even creating their own personalized platforms.

With regards to CTC technologies, Ryan Dittamore (Epic Sciences Inc., San Diego, CA, USA) spoke of the “no cell left behind” method developed and utilized by Epic Sciences. This approach does not rely on antigen-based CTC enrichment, but instead lysed whole-blood is spun onto microscope slides for long term storage, enumeration, or subsequent downstream interrogation using a variety of amenable methods (e.g., immunofluorescence staining, fluorescence in situ hybridization (FISH) analysis, NGS). The capacity for extremely high-resolution imaging on this platform has allowed for the identification of a number of distinct CTC subtypes shown to be associated with both disease stage and therapy response [19]. Likewise, Shana Kelley (University of Toronto, Toronto, ON, Canada) discussed the development of a novel microfluidics-based platform in her laboratory capable of detecting, collecting, and isolating CTC subpopulations with variable EpCAM expression. This platform is highly adaptable and amenable to different capture antigens and on-chip or downstream analysis techniques. Using this technology, Kelley’s lab has begun to investigate the significance of alterations in the EpCAM profile over time in both patient samples and pre-clinical experimental models of cancer [20,21]. Both Susan Done (University of Toronto, Toronto, ON, Canada) and Sabine Mai (University of Manitoba, Winnipeg, MB, Canada) have employed commercially available technologies using immunomagnetic (Miltenyi Biotec, Cologne, Germany) and filtration techniques (ScreenCell, Westford, MA, USA), respectively, for enrichment. Following separation, both investigators used downstream techniques for genomic assessment, with Done investigating the identification of a CTC genomic signature in advanced breast cancer patients [22] and Mai assessing prostate cancer CTCs for 3D nuclear telomere organization, a proposed surrogate for genomic instability at the single-cell level using a software technology developed in her laboratory called TeloView [23]. Results from all investigators suggest that CTC heterogeneity and/or genomic instability may act as a surrogate biomarker for more unstable/aggressive disease and ultimately treatment resistance.

Among the speakers presenting recent cfDNA research, one common theme reiterated throughout all talks was the need to move beyond the study of single gene mutations, and to develop and validate large multi-gene panels to capture a more comprehensive snapshot of the tumor genome. These gene panels would most likely need to be optimized for particular disease sites but in theory, once developed, these similar approaches could be utilized across of number of different cancers. Workshop Co-Chair Alexander Wyatt (University of British Columbia, Vancouver, BC, Canada) described a custom 72 “prostate cancer driver gene” panel for screening patients with castration-resistant prostate cancer (CRPC) for clinically-relevant mutations and copy number changes that inform on therapy-response and could potentially be exploited to stratify patients for distinct treatment protocols [24,25,26,27]. Wyatt’s approach relies on deep NGS of isolated cfDNA, using a capture-based technique. He noted that this simple approach (without the use of molecular barcodes described below) holds the most potential in patients with metastatic disease where the ctDNA fraction of total cfDNA is frequently >1%. In the future, Wyatt anticipates that screening prostate cancer patient cfDNA will be particularly helpful for prognostication and therapy response prediction given the logistical barriers to obtaining tissue biopsies in a bone-predominant disease, but that technology advancements may also aid diagnoses as well. Ryan Morin (Simon Fraser University, Burnaby, BC, Canada) described his reasoning for selection of a digital droplet PCR (ddPCR) approach to investigate the genetic evolution of non-Hodgkin’s lymphoma, specifically diffuse large B-cell lymphomas following treatment relapse [24,25,26]. During this stage of disease, repeated tissue/bone marrow biopsies may be infrequent due to the high rate of disease progression. Morin explained that ddPCR analysis of cfDNA demonstrated significant advantages, in that it allowed for high sensitivity read-out using a multi-marker panel, but was disadvantaged by its need to target specific, previously identified/characterized mutations [28]. He introduced a gene panel-based sequencing strategy with molecular barcoding for error correction that allows accurate detection of many mutations in cfDNA from lymphoma patients. Use of this methodology in a recently published clinical trial in diffuse large B-cell lymphoma was shown to be a powerful early measure of treatment efficacy [29]. Finally, Scott Bratman (University of Toronto, Toronto, ON, Canada) provided an excellent clinician-scientist’s perspective on the field, focusing mainly on the benefits and limitations of using either PCR- or NGS-based approaches for analyzing cfDNA [30,31,32]. Bratman suggested that the high-sensitivity and low cost of PCR-based approaches make them advantageous (compared to the higher costs and longer-processing times associated with NGS) but that they are still limited by their need for significant protocol optimization and known mutational targets for analysis.

It was notable that even at this small and focused Workshop, there was tremendous diversity in terms of technology/technique selection utilized by researchers in their laboratories. These selections are driven not only by research needs, but also by available resources, and cost considerations. Although this diversity may aid in the identification of individual platform weaknesses and ultimately improve next-generation protocols, it considerably limits the ability to interpret and compare results across laboratories. To further complicate the field, there has been little investigation into the overlapping information that CTCs/cfDNA provide. Are these biomarkers equivalent, complementary, and/or do they serve distinct purposes, providing unique prognostic/predictive information? Importantly, the clinical scenarios in which one or both biomarkers should be used and research to support these findings is needed and therefore was an important topic for discussion at the Workshop.

## 3. Theme 2: CTCs versus cfDNA: Comparable or Complimentary Biomarkers?

**Figure 1:**
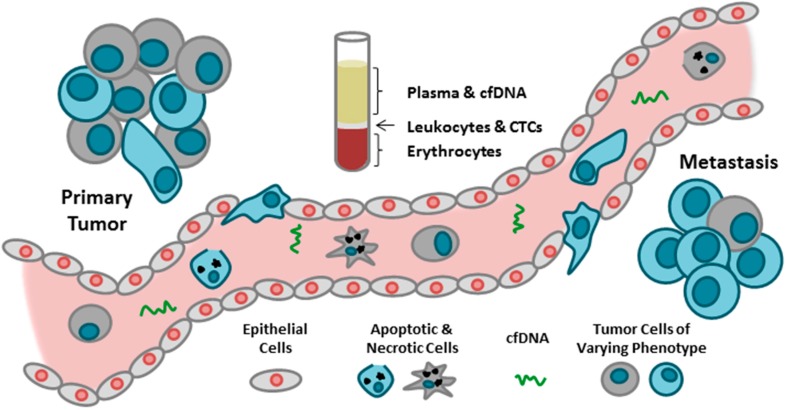
Comparison of circulating tumor cells (CTCs) and cell-free DNA (cfDNA) as blood based biomarkers in oncology.

Over the past decade, growing evidence has supported the potential of CTCs and cfDNA as circulating biomarkers that could act as “liquid biopsies”, providing information about disease progression and therapy response in real-time. The blood serves as a reservoir of CTCs and cfDNA derived from both primary and metastatic sites. These rare biomarkers are easily accessible using minimally invasive venipuncture, allowing for repeated blood sample collection and therefore repeated “biopsy”, which is not always feasible when performing tissue biopsies. In addition, both CTCs and cfDNA have been demonstrated to provide prognostic information in specific tumor types and disease settings depending on the number/level detected in collected blood samples [11,12,13,33] (Figure 1 and Table 1).

Although these biomarkers have been proposed to have comparable/equivalent potential utilities (e.g., risk assessment for metastatic relapse/progression, patient stratification, real-time monitoring of therapies, identification of therapeutic targets and resistance mechanisms), further investigation is required to determine if one biomarker is superior to the other depending upon the clinical scenario in which they are being utilized [6]. This topic was discussed in the opening presentation by Workshop Co-Chair Alison Allan in which the biological, analytical, and technological differences between CTCs and cfDNA were identified and presented to the workshop attendees as important considerations before incorporation of these biomarkers into clinical practice (Figure 1, Table 1). Specifically, Allan highlighted the origins of both CTCs and cfDNA; with CTCs derived as intact cells shed from the primary or metastatic tumor sites (which may or may not be viable), versus cfDNA, which is likely derived from either cells that are actively shedding cfDNA or cells that have undergone apoptosis/necrosis [5,35]. In addition, considerations regarding the potential downstream analyses that could be performed following isolation of each biomarker were outlined, including DNA, RNA, and protein for CTCs, compared to DNA only for cfDNA. Recent advances have also allowed for culture of isolated CTCs, giving them the added advantage of being further interrogated using both in vitro and in vivo functional assays. This approach in particular holds great promise for the extensive assessment of therapeutic efficacy [38]. Finally, the technological requirements for CTC and cfDNA assessment were reviewed, with both biomarkers requiring exquisitely sensitive detection techniques, but with CTCs also requiring specialized instrumentation for capture. The issue of cost was also briefly addressed by Allan and mentioned as a consideration for appropriate biomarker selection and potential limitation for clinical implementation.

Many of the other presenters proposed optimal clinical settings in which CTCs and/or cfDNA would have the greatest potential benefit. Alexander Wyatt discussed using cfDNA for identifying actionable/druggable target mutations for individualized patient care. He also noted that cfDNA has the added advantage of being easily collected and stored for analysis at a later date, which may provide benefit in both the clinic as archived specimens and as a cfDNA bank for future research initiatives. Wyatt also noted that in the future cfDNA may be the most efficient tool for detecting low levels of disease and therefore may be valuable in monitoring patients and even for improved diagnosis. This idea of low level detection using cfDNA was further echoed by Scott Bratman. Bratman pointed out that one of the most promising applications of cfDNA analysis is for detection of minimal residual disease after treatment, particularly for patients in curable stages of disease who have received definitive treatment and for whom adjuvant treatment may be considered [42,43,44,45,46]. The utility of cfDNA-based minimal residual disease detection in nasopharyngeal carcinoma is currently being tested in an international biomarker-stratified randomized trial (NCT02135042) [47]. Morin also indicated that recurrent genetic changes resulting from clonal evolution can be found in relapsed patients and such variants may be detected in cfDNA using targeted assays, thereby detecting acquired treatment resistance earlier than current methods. It is noteworthy that one potential limitation to the utility of ctDNA is the cells from which it originates. If the DNA utilized for analysis originates from apoptotic/necrotic CTCs, these results may have limited applicability for determining therapeutic resistance genes/mechanisms. However, if the DNA instead originates from actively shedding tumor cells (either in circulation or in primary/metastatic sites) these results could provide extensive resistance profiles. However, these relationships still need to be assessed in large clinical trials. Several presenters noted that CTCs, unlike cfDNA, can be assessed at the single cell level. While single-cell assessment allows for in-depth investigation of the clonal evolution of disease, it also demonstrates the true level of heterogeneity that may be present both between patients and within an individual patient, thereby further complicating interpretation of CTC characterization and cfDNA mutation analysis. The promise of, and problems associated with disease heterogeneity were an important topic discussed throughout this workshop.

## 4. Theme 3: Importance of Heterogeneity

The presence of disease heterogeneity in cancer is not a new concept. In fact, investigation of this heterogeneity is routine practice across a variety of tumor types and is implemented for the selection of appropriate treatment regimens (e.g., hormone receptor expression in breast cancer). Disease heterogeneity is the main reason why some patients will respond quickly and dramatically to a particular treatment while others will experience no benefit [48]. This heterogeneity occurs at multiple levels: between and within tumor types, between and within individual patients, during the transition from primary tumors to metastatic lesions, and even within the same lesion there can be cells with dramatic differences in gene expression [2,49,50,51]. Based on this data, it should therefore be no surprise that protein and/or nucleic acid characterization of CTCs and mutational analysis of cfDNA reflects a similar degree of heterogeneity, with dramatic differences observed both between patients, and within the same individual [39,52,53]. This idea was reinforced by Alexander Wyatt, who reported on a study conducted in his laboratory in which mutations observed in cfDNA liquid biopsies were compared to solid biopsy specimens from the same patient. The results demonstrated that within some patients the mutations observed in tissue biopsy samples matched well with those seen in the cfDNA, while in others there was poor concordance between specimens. 

It has been proposed that the use of cfDNA/CTCs would be most beneficial for clinical settings in which the collection of tissue biopsies is either not advised or not feasible. In contrast to tissue biopsies, liquid biopsies are minimally invasive and can be performed routinely. These potential “surrogate biopsies” hold promise to provide information about disease progression in real time, thus informing treatment regimens and ultimately improving patient outcomes. Recently, characterization of nuclear AR-V7 protein in the CTCs of mCRPC has demonstrated a statistically significant therapy interaction between the biomarker and improved overall survival with taxane chemotherapy over AR signaling inhibitors in multivariate models, supporting potential predictive clinical utility and may identify one of the first predictive cfDNA/CTC tests [37].

Another approach described by many of the presenters at this workshop was utilizing the overall disease heterogeneity, instead of specific characteristics/detected mutations, to risk-stratify patients into high-versus low-risk prognostic groups. Dittamore and others described the use of the Shannon Index [54] to obtain a quantifiable measure of disease heterogeneity within an individual based on morphological heterogeneity of CTCs. Using this approach, his group was able to demonstrate that CTC heterogeneity was associated with poor patient outcomes [55,56]. This hypothesis was further supported by work from Susan Done’s laboratory when considering heterogeneity in disease aggressiveness in breast cancer and Shana Kelley’s work examining heterogeneity in EpCAM profiles of CTCs. In addition, Dittamore suggested that this observed heterogeneity may result from selective treatment pressure, which in concert with genomic instability could lead to rapid changes in the genotype of CTCs, thereby potentially contributing to treatment resistance and disease progression.

The results presented here suggest that, specifically when considering CTCs, single-cell analysis may be an optimal approach. However, this is still technologically challenging and single cell genome analysis, depending on the number of CTCs, can be tremendously expensive. Interestingly, Dittamore’s research suggests that the cellular phenotype of CTCs shows concordance with mutations seen in the genome of these cells and therefore cellular phenotyping may be sufficient in many circumstances. Assay expense is a necessary consideration before incorporation of these assays into a clinical setting. Many questions have arisen regarding the most cost-effective, yet informative way to obtain information that can then be utilized to augment patient care. The optimal assay must be selected and, as has already been suggested, this will very likely differ across disease type, and stage.

## 5. Theme 4: Considerations for Incorporation of CTCs and cfDNA into Clinical Trials

Despite the fact that significant evidence exists to support the use of CTCs for prognostication, there has not been widespread utilization of these techniques in the clinical setting [11,12,13]. Additionally, although cfDNA has just recently reached the level of standardization necessary for clinical implementation, we anticipate there will be a similar delay in the use of these methods as well. Scott Bratman addressed this lack of utilization during his lecture and suggested that the disappointing results from the highly anticipated SWOG0500 trial (switching versus maintaining chemotherapy regimens based on changes in CTC number), highlight the difference between prognostic and predictive biomarkers [57]. He further emphasized that prognostic biomarkers must be able to significantly add to the information obtained from other validated clinical/pathological features and that physicians need to be able to act on this additional information in order for it to be of value. Bratman also noted that even if these prognostic biomarkers provide this additional information, physicians need to have the tools/treatments to address this elevated risk, which unfortunately is very often not the case. Second and third line therapies often provide little benefit to the patient, making proper interpretation of trial results involving these patients difficult.

Several presenters provided additional suggestions for clinical trial design and trial implementation. Alexander Wyatt noted that it is important to remember that most studies of CTCs and cfDNA have been carried out in retrospective cohorts, often without enough regard to patient stage, treatment line, etc. Therefore, he suggests that the first logical step is to examine the same questions in the context of ongoing clinical trials where blood collection is mandatory—this will help improve the impact of results, and allow extension, interpretation, and generalization to other similar matched patient cohorts (NCT02125357 and NCT02254785) [47]. The second step would then be the rational design of prospective clinical trials to evaluate the biomarkers, likely using an umbrella design. Ryan Dittamore recommended that trials be driven by clinical decisions rather than biomarker-driven, noting that there must be strong association between the potential biomarker of interest and the clinical decision point. Additionally, he reminded workshop attendees to be generous with the number of samples collected (“you will always need more samples than you think you will”). This will help avoid selection bias as well as broadening the applicability of results, since cancer is always more complex/heterogeneous than anticipated and this should be accounted for in trial design. As previously mentioned, these trials are often performed with less than ideal patient populations; therefore, Scott Bratman suggested that clinical trials involving CTCs and cfDNA be performed with tools/treatments that we know are effective in order to obtain “real” results. Additionally, Bratman noted that one must consider that the tools/treatments applied to your study group may dramatically reduce the detectability of your biomarker, especially if its presence is low to begin with. This can make it exceptionally difficult to discriminate responders from non-responders in your patient population. Additionally, one must have rigorous standards for controlling pre-analytical variables related to sample collection, processing and banking. In addition, it is important to consider the timing of sample collection and what information is important to obtain at which time points (e.g., deciding when enumeration is sufficient or when characterization is necessary). Finally, although recent technological advances have allowed for easier enrichment and detection of CTCs and cfDNA, we must not forget that these biomarkers are still considered rare events. This rarity further emphasizes the need for appropriate standardization and validation of pre-analytical and analytical SOPs in order to yield quality results that are highly reproducible.

Above and beyond the unmet needs described throughout this Report and the detailed considerations for future clinical trial design, arguably the greatest challenge facing the field is its vast diversity. In recent years, there has been a surge in the development and commercialization of novel technologies aimed at CTCs and cfDNA enrichment and detection. Unfortunately, this overwhelming number of technologies has led to a similarly overwhelming number of published clinical trials using inconsistent technologies, thus preventing direct head-to-head comparison and difficulty in interpreting clinical trial results. This lack of consensus in technology selection, as well as lack of consistent data across platforms will continue to be a challenge in the field unless these considerations and comparisons are included as primary objectives in the design of future trials. Although no formal recommendations regarding technology selection were made at the conclusion of the meeting, it was agreed that the ideal technology would have high capture efficiency, produce material that is highly amendable to downstream analysis, allow for the isolation and interrogation of single cells (CTCs), and be highly reproducible.

## 6. Conclusions

The field of circulating biomarkers is ever-growing and changing in the face of novel technological advancements, improved translational research, and clinical trial development. However, we still face challenges with regards to the interpretation of data obtained from these assays and how to utilize these results to improve patient treatment and outcomes. Therefore, more workshops such as these are necessary to address and discuss setbacks and successes in the field and to fuel meaningful collaborations and partnerships between researchers and clinicians. The lectures presented at this year’s Workshop show great progress and promise in the field (summarized in Table 2), despite ongoing challenges. These advances made in both basic and translational research will ultimately impact patient management and outcomes.

## Figures and Tables

**Table 1:** Summary comparison of circulating tumor cells (CTCs) and cell-free DNA (cfDNA) *.

Comparison	CTCs	cfDNA
Origin	Intact cells (not necessarily viable) [34]	Necrotic/apoptotic cells [33] and/or actively secreted from intact cells [35]
Definition	Tumor cells derived from primary/metastatic sites [6]	Fragmented DNA in circulation [6]
Capture & Analysis Techniques	Enrichment: size/density-, immunomagnetic-, or microfluidic-based [10] Detection: protein- or nucleic acid-based [10]	Enrichment: plasma collection [33] Detection: PCR-, or sequencing-based [33]
Advantages	Extensive downstream analysis (DNA, RNA, protein, functional assays) [22,36,37,38] Assessment of single cells [39] Clinically-validated technology available (CellSearch^®^ system; metastatic breast, prostate, & colorectal cancers) [11,12,13] Captured viable cells can be used for in vitro culture or in vivo animal studies [38]	Easy to isolate/enrich from whole blood [33] Amenable to long-term storage for subsequent analysis [33] High-sensitivity read-out [28] Clinically validated test for EGFR mutations in non-small cell lung cancer [9,40]
Disadvantages	Low cell numbers in non-metastatic setting [41] Challenging to store long-term and subsequently analyze (Lowes, L.E., unpublished) Both detection and enrichment steps require highly sensitive and often expensive technology [6]	Limited (pre-)analytical/analytical SOPs, assay validation, & appropriate prognostic/predictive read-out (may be disease/mutation specific) [33] Limited downstream analysis (DNA only) Currently only feasible in high tumor burden setting [6] Need known target mutations to confirm cfDNA originated from tumor cells [6]

* For a comprehensive review of CTCs and cfDNA please see the following excellent review articles [5,7,8].

**Table 2:** Summary of Workshop Findings: Implications to the Field of circulating tumor cells (CTCs) and cell-free DNA (cfDNA) Biomarkers.

**Recent Technological & Methodological Advances**
No enrichment approaches: - EPIC Sciences unique “no cell left behind” approach allows for detection of CTCs without an initial enrichment step typical for other methods. Identification and isolation of CTC subpopulations: - Both the technology developed by EPIC Sciences and the microfluidic approach described by Shana Kelley described the ability to identify and isolate CTCs from distinct CTC subpopulations (e.g., EpCAM expression). Analysis of these distinct populations holds promise for understanding disease biology. Assessment of CTCs at the single-cell level: - Recent improvements in technology have begun to allow for the isolation and interrogation of individual CTCs. These advances provide opportunities to assess overall disease heterogeneity, a commonly discussed theme at this workshop. Development of multi-marker gene panels for assessment of cfDNA: - A commonly discussed theme throughout this workshop was shift in focus from single-gene assessment to multi-marker panels capable of extensive genomic mutation and copy number change assessment, thus providing a more comprehensive overview of disease.
**Considerations for Incorporation of CTCs and cfDNA into Clinical Trials:**
Trials should be clinical decision-driven not biomarker-driven, with appropriate biomarkers significantly adding to the prognostic and/or predictive information already obtained via validated methods. In addition, physicians need to have the appropriate tools to address this elevated risk. Trials need to be performed in disease settings with tools and treatments that are known to be effective in order to appropriately assess the value of CTC/cfDNA to treatment efficacy and disease outcome. When designing trials, investigations must consider the impact of *treatment* itself on the biomarker of interest. It is likely that the biomarker load will significantly decrease following treatment, making assessment of its true value difficult or even impossible. Special consideration must be placed on designing, implementing and validating standard operating procedures (SOPs) for the collection and analysis of samples. Appropriate selection of the timing of sample collection is critical, and should be based on the specific biology of each disease (e.g., baseline, throughout treatment, following treatment completion, and follow-up samples). Must determine if characterization is necessary or if enumeration will suffice. If characterization *is* deemed necessary, one must then decide at what level the collected sample will need to be assessed (DNA, RNA, protein, functional assays) to properly answer the posed question(s). Technology selection is important, especially with regards to previous trial data, and widespread feasibility based on overall cost must be considered. Appropriate statistical evaluation of the number of patients required to answer posed questions. Typically, larger number of patients will be required than standard clinical trials due to the rare nature of CTCs/cfDNA and overall disease heterogeneity. Most importantly, how results will be analyzed and interpreted, and if the obtained data can be compared head-to-head with previously performed or ongoing clinical trials.
**Moving Forward: General Considerations for the Future Use of CTCs and cfDNA**
CTC and cfDNA analysis should be incorporated into ongoing clinical trials where blood collection is mandatory, thus allowing for greater generalizability and more impactful results. Need to develop SOPs for cfDNA and CTC sample archiving, and make this routine practice for ongoing clinical trials, thus allowing reassessment or further assessment of archived samples following technological advances. Need to design trials that incorporate *both* CTCs and cfDNA to allow for direct comparison and determination of each biomarker’s role and value in various disease settings. Need rationally designed *prospective* trials from which to draw meaningful conclusions.
